# The Real Hardship of the Medical Profession from Military Service

**Published:** 1918-12

**Authors:** 


### The Real Hardship of the Medical Profession, from Military Service.

Instead of indulging in general expressions of sympathy with ourselves, let us get down to the gist of the matter and concentrate on an effort to secure such relief as is really needed. The medical profession may be heroic, stoic or mercenary but the only serious apprehension that has been expressed sincerely and repeatedly, is as to the financial condition of the medical reserve officer when the war is over. It is not the fear of being killed or even of being mutilated or blinded, nor the difficulty of living and supporting a family on the pay of even a first lieutenant during the war that troubles the medical profession, but the re-establishment of a practice after the war. For reasons that need not be discussed for members of the medical profession, this re-establishment is a much more serious matter than for the man in any capacity who has simply given up a job and who expects always to support himself by a job of one kind or another. It is more serious than for men having independent businesses, unless they are the sole managing force in that business and men thus circumstanced will probably be exempt. It is a problem that is automatically met, at least to a large degree, for teachers, clergymen, engineers and many other professional men leaving their ordinary employment for military service.

The problem, even for the medical profession can be reduced by the elimination of certain factors. Granting that the war lasts only for about six months, even a little longer, one need not worry much about re-establishment of practice. Experience has shown over and over again that an absence of this duration, taken as an occasional vacation, a few times during one's life, or for post graduate study, or any other valid reason, does not materially injure a practice. We can eliminate the men, with or without dependents, who have not really established a practice. They have to undergo this ordeal anyway and they will have an easier time with the experience and prestige of military service and the savings which they ought to make from their pay, than under ordinary peace conditions. We can also eliminate the men of extraordinary reputation who, whatever they have sacrificed during the war, will return to peace practice with added experience and prestige. And, of course, we can eliminate those —a small minority of the profession—who happen to be endowed from any source, with sufficient means to render them wholly or mainly independent of practice.

The problem is thus reduced to men of various ages between 30 and 50, as approximate limits, mainly with dependents—and a man without dependents can always get along somehow—whose average professional earnings correspond fairly well to the range of pay from first lieutenant to major, who have good professional reputations and established practices, but who are not famous, and whose living has not depended on the particular lines of practice which will be especially benefitted by military experience. The problem will be especially hard for men still far short of the retiring age in peace but near enough to it so that they will be liable to a considerable degree of physical deterioration from military service and who, even in the absence of such deterioration, will be regarded as out of place in the struggle of building up a practice.

We have no faith in the theory of holding together the practice of the absent physician. Permanent family clienteles are rare today, except in very small villages from which medical officers will be drawn only in exceptional cases, or persisting for a few of the older physicians in cities, these men being in most instances beyond the age limit of service. It is very easy to formulate a scheme for preserving intact the practice of a physician absent on military duty and we do not question but that the profession would undertake such a scheme in good faith. But it will fail, first because such a practice is the exception, secondly because when it exists, its individual components can not be endorsed over to some one else, like a check.

The only practical ways of meeting the problem that we can suggest are these:

1. After six months' service, give the medical reserve officer the same privilege of retiring on reduced pay that is accorded to members of the regular Medical Corps, but with rigid insistance on adequate physical reasons.

2. After the same length of service, give him the same permanence of appointment, provided his services are satisfactory. If they are not regarded as satisfactory for permanent employment but as sufficiently good in an emergency, give him the choice between immediate resignation and a continuance which he definitely understands to be at the option of the government.

Permanent employment does not by any means signify lifelong service. Any officer is free to resign unless his services are needed at the time. It is scarcely probable that any overwhelming number of medical reserve officers, at the end of of 2-5 years service, would desire to stay in the service. Even if 10,000 remained, as a dead-weight on the hands of the government, the expense to the government would be only about $25,000,000 for the first year and this amount would be rapidly diminished by death and voluntary resignation. If we establish universal military-training or anything approaching it after the war, a large permanent medical corps would be required. If not, at least by co-operation with the state governments, some form of useful work can be found for the largest number of medical officers conceivable as desiring to remain in government employ. Such work would easily be worth to the country, the $25,000,000 estimated.

